# Dual Behavior of Long-Chain Fatty Acids and Their Cyclooxygenase/Lipoxygenase Metabolites on Human Intestinal Caco-2 Cell Growth

**DOI:** 10.3389/fphar.2020.529976

**Published:** 2020-09-04

**Authors:** Carolina E. Storniolo, Marisol Cabral, Maria A. Busquets, Raquel Martín-Venegas, Juan J. Moreno

**Affiliations:** ^1^Department of Nutrition, Food Sciences and Gastronomy, Faculty of Pharmacy and Food Sciences, University of Barcelona, Barcelona, Spain; ^2^Institute of Nutrition and Food Safety (INSA-UB), University of Barcelona, Barcelona, Spain; ^3^Department of Pharmacy, Pharmaceutical Technology and Physical-Chemistry, Faculty of Pharmacy and Food Sciences, University of Barcelona, Barcelona, Spain; ^4^Institute of Nanosciences and Nanotechnology, University of Barcelona, Barcelona, Spain; ^5^Department of Biochemistry and Physiology, Faculty of Pharmacy and Food Sciences, University of Barcelona, Barcelona, Spain; ^6^CIBEROBN Fisiopatología de la Obesidad y Nutrición, Instituto de Salud Carlos III, Madrid, Spain

**Keywords:** colorectal cancer, eicosapentaenoic acid, hydroxyeicosapentaenoic acids, oleic acid, prostaglandin

## Abstract

Etiology of colorectal cancer (CRC) is related, at least in part, with nutritional profile and epidemiological data indicating a key role of dietary fat on CRC pathogenesis. Moreover, inflammation and eicosanoids produced from arachidonic acid might have a pivotal role in CRC development. However, the effect of specific fatty acids (FAs) on intestinal epithelial cell growth is not completely studied now. By this reason, the aim of this work is to unravel the effect of different saturated and unsaturated long-chain fatty acids (LCFA) and some LCFA metabolites on CRC cell line growth and their possible mechanisms of action. Our results demonstrated that oleic acid is a potent mitogenic factor to Caco-2 cells, at least in part, through 10-hydroxy-8-octadecenoic synthesized by lipoxigenase pathway, whereas polyunsaturated FAs such as eicosapentaenoic (EPA) acid has a dual behavior effect depending on its concentration. A high concentration, EPA induced apoptosis through intrinsic pathway, whereas at low concentration induced cell proliferation that could be related to the synthesis of eicosanoids such as prostaglandin E_3_ and 12-hydroxyeicosapentaenoic acid and the subsequent induction of mitogenic cell signaling pathways (ERK 1/2, CREB, p38α). Thus, this study contributes to understand the complicated relationship between fat ingest and CRC.

## Introduction

Cancer causes around 7 million of deaths annually, becoming 12.5% in the entire world and colorectal cancer (CRC) is the third leading cause of cancer-related death in developed countries (Ullman and Itzkowitz, 2011). According to recent rates, the lifetime risk of developing CRC is 4.3% (NCI). Although a great effort has been made toward developing detection and surgical strategies, there has been little improvement in the outcome for patients with advanced disease.

CRC is linked with environmental factors, being lifestyle and nutritional profile the major but controllable implicated factors. In fact, around 90% of the CRC cases appear to be related to lifestyle, with the highest incidence in economically developed countries (Clinton et al., 2020). Refined carbohydrates, alcoholic beverages, red and processed meat, saturated fat, and a high energy intake, specially associated with abdominal body fatness, would favor CRC development. In contrast, high consumption of dietary fiber, fruits and vegetables, calcium, antioxidants, and vitamins would have the opposite effect (Meyerhardt et al., 2007; WCRF, 2011). Thus, westernized diet pattern is associated with an increased risk for CRC (Yusof et al., 2012).

According to the World Health Organization the average global intake of fat has increased over the last half-century (WHO). However, not all fats are the same and it is now well established that saturated, *trans* fats and unsaturated fats can act in opposite ways to influence human health, including promotion of cancer. Thus, the data indicated that not only the amount of dietary fat but also fat diet composition could be determinant in the pathogenesis of different neoplasms as CRC (Barone et al., 2014). Some experimental studies demonstrated that ω-3 PUFA protects against CRC, while ω-6 PUFA promotes this cancer development, proposing mechanisms like modulation of inflammation, cellular oxidative stress, membrane dynamics, and cellular receptors function (Larsson et al., 2004; Cockbain et al., 2012). However, in epidemiological studies there are many different results, and data seem to be inconclusive regarding the effect of these FAs on CRC. Some authors demonstrated an inversely relation between ω-3 or ω-6 PUFA consumption and CRC risk (Chao et al., 2005; Norat et al., 2005), others indicated a lack of association (Sasazuki et al., 2011; Song et al., 2014), while some shown a positive relationship (Daniel et al., 2009; Shen et al., 2012). In the other hand, studies including saturated fat consumption and CRC, demonstrated that there is a direct relation between these two factors (Giovannucci and Goldin, 1997; Rosignoli et al., 2008) but other authors have not found any significant relation (Williams et al., 2010. Oleic acid, ω-9 FA, is considered one of the healthier sources of fat in the diet. Also, oleic acid was attracted much attention as characteristic component of Mediterranean diet and has been linked to a protector effect against cancer (Escrich et al., 2007).

Eicosanoids produced from arachidonic acid (AA) might have a pivotal role in these events. AA biosynthesized from linoleic acid is the substrate of cyclooxygenase (COX) and lipoxygenase (LOX) for the production of eicosanoids such as 2-series prostaglandins (PGs), 4-series leukotrienes (LTs) and HETEs that may facilitate CRC progression by stimulating cell proliferation and survival, tumor cell invasiveness, and angiogenesis (Moreno, 2005; Moreno, 2009). PUFAs such as eicosapentaenoic acid (EPA) can also be released from cell membrane phospholipids and can act as substrate for COX and LOX pathways, giving rise to the 3-serie PGs, 4-serie LTs, and hydroxyeicosapentaenoic acids (HEPEs) (Smith, 2005) which effects on cell proliferation are poorly known.

Considering all together, the effect of FAs on intestinal epithelial cell growth is not completely understood now. By this reason, the aim of this study is to determine the effect of representative saturated and unsaturated long-chain FAs (LCFA) and some fatty acid metabolites on CRC cell line growth and their possible mechanisms of action.

## Material and Methods

### Materials

Dulbecco’s modified Eagle’s medium (DMEM), trypsin/EDTA, penicillin, and streptomycin were supplied by GIBCO (Paisley, Scotland, UK). Acridine orange, bovine serum albumin (BSA) de-fatty BSA, carbonyl cyanide 3-chlorophenylhydrazone (CCCP), Dulbecco’s PBS, ethidium bromide, fetal bovine serum (FBS), nonessential amino acids, paraformaldehyde extra pure, propidium iodide, ribonuclease A from bovine pancreas, SC19220, tetramethylrhodamine ethyl ester perclorate (TMRE), and Triton X-100 were supplied by Sigma-Aldrich (St. Louis, MO, USA). 10-hydroxy-8-octadecenoic acid (10-HODE) was from Parchem (New Rochellem, NY, USA). Tissue culture supplies and sterile material were obtained from Corning, Nirco S.L., NORM-JECT and Biosigma S.R.L. (Italy). The BioRad Protein Assay was obtained from Bio-Rad Laboratories, Inc. Myristoleic, palmitoleic, stearic, oleic, elaidic, linoleic, α-linolenic, γ-linolenic, mead, arachidonic (AA), EPA, erucic and DHA acids, prostaglandin (PG) E_2_, PGE_3_, leukotriene (LT) B_4_, LTB_5,_ 12-S-HETE, 12-S-HEPE, ketoprofen, baicalein, MK571, MK886 LY171883, zileuton, U75302, and the PPARγ ligand screening assay were supplied by Cayman Chemical Co. (Ann Arbor, MI, USA). Cell proliferation ELISA–BrdU (Colorimetric) Kit was from Roche (Basel, Switzerland). LY255283 was from Tocris Biosc. (Bristol, UK). AH23848 and ONO-329 were kindly provided by Glaxo-Wellcome (Stevenage, UK) and Ono Pharmaceutical Co. Ltd. (Osaka, Japan), respectively. The MebStain Apoptosis Kit was supplied by MBL International (Woburn, MD, USA) and the Multi Kinase Array (MKA) ElA by Symansis (Auckland, New Zealand).

### Cell Culture

Caco-2 cells were derived from a moderately well-differentiated primary colon adenocarcinoma and were provided by American Type Culture Collection (HTB-37) (Manassas, VA, USA). The cells were routinely grown in 25 or 75 cm^2^ plastic flasks at a density of 1 × 10^4^ cells/cm^2^ and cultured in DMEM with 4.5 g/L D-glucose and 2 mM L-glutamine, and supplemented with 1% (v/v) nonessential amino acids, 10% (v/v) heat-inactivated FBS, 100 U/ml penicillin, and 100 µg/ml streptomycin. Cells were incubated at 37°C under a humidified atmosphere of 5% CO_2_ in air. Cells grown to ~80% confluence were released by trypsinization and subcultured at a density of 1.5–2 × 10^4^ cells/cm^2^ in 12 mm diameter plastic clusters and of 1 × 10^4^ cells/cm^2^ in 60 mm diameter plastic dishes. Growth medium was replaced twice per week. Although cancerous in origin, Caco-2 cells undergo a gradual differentiation process that takes place spontaneously once confluence has been reached and that is completed after 21–25 days in culture (Martín-Venegas et al., 2006). Considering these, the experiments were performed in cells maintained for 3 days in culture to use undifferentiated Caco-2 cell cultures to perform all experiments. All experimentation products were diluted in DMSO (final concentration of DMSO was lesser than 0.1%). FAs stock solutions prepared in DMSO were further diluted in cell medium supplemented with FBS or cell medium containing 5% de-fatty BSA as a carrier to ensure FAs dissolution. Solutions containing equal amounts of de-fatty BSA were used as control.

### Cell Growth and DNA Synthesis Assays

The effect of the treatments was assessed on Caco-2 cells clusters in 24-well plates (5–10 × 10^3^ cells/cm^2^). Cells were cultured for 96 h in DMEM supplemented with 10% FBS. Then, cells were incubated for 48 h in the presence of treatments. Finally, cells were washed, trypsinized, and counted under a microscope using ethidium bromide/acridine orange staining to count viable cells.

DNA synthesis in Caco-2 cells was assessed by a colorimetric immunoassay based on the measurement of BrdU incorporation during DNA synthesis (Cell proliferation ELISA, BrdU Kit, from Roche). Caco-2 cells were cultured at 1,000–1,500 cell/well in 96 well plates for 96 h in DMEM supplemented with 10% FBS. Then, cells were incubated for 48 h in the presence of compounds. Thereafter, cells were treated following the manufacturer instructions. Absorbance was measured at 450 nm in a plate reader (TECAN, Sunrise, Grödig, Austria).

### TUNEL Assay

Degradation of chromosomal DNA was evaluated with TUNEL method using a MebStain apoptosis kit (MBL Int.). After 96 h in culture, Caco-2 cells were cultivated in media containing treatments for 48 h. Next, cells were fixed with 4% paraformaldehyde and permeabilized with 70% ethanol. Thereafter, 3′-OH DNA ends generated by DNA fragmentation were labeled with fluorescein-dUTP, mediated by terminal deoxynucleotidyl transferase, and were analyzed on an Epics XL flow cytometer (Coulter Corporation, San Francisco, CA).

### PGE_2_ and PGE_3_ EIA Determination

PGE_2_ and PGE_3_ concentrations were determined in the Caco-2 culture medium. Cells were overnight with FBS or FBS plus EPA (10 μM). Two hundred fifty μl aliquots of the culture medium were acidified with 1 ml of formic acid and PGs were extracted in ethyl acetate. After discarding the aqueous phase, the organic phases were evaporated under a stream of N_2_ and PGE_2_ or PGE_3_ were determined with a monoclonal enzyme immunoassay kit (MyBiosource, San Diego, CA) according to the manufacturer’s protocol.

### Measurement of Cell Signaling Pathways

Cells were seeded in 60 mm plastic clusters (10^4^ cells/cm^2^). After 4 days, the cultures were incubated with the treatments in absence of FBS. Maximal phosphorylation was observed after 5 min incubation for ERK1/2, Akt, and p38, and after 15 min for GSKβ and CREB. To measure the kinase activation with total cellular lysates, Caco-2 cells were lysed using a denaturing cell lysis buffer containing 6 M urea and protease (leupeptin 2 µg/ml, pepstatin 10 µM, aprotinin 3 µg/ml) and phosphatase (NaF 5 mM, Na_4_P_2_O_7_ 2 mM, Na_3_VO_4_ 1 mM) inhibitors. The resulting solutions containing 80–100 µg of proteins were then added to a kinase ELISA plate and the assay was performed following the manufacturer’s recommendations (Symansis). Finally, optical density was measured at 450 nm. Thus, we studied the effect of PGE_2_/PGE_3_ on the phosphorylation of AKT1 (pS473), AKT2 (pS474), ERK1/2 (pT202/Y204; pT185/Y187), GSK3β (pS9), p38α (pT180/Y182), and CREB (pS133).

### PPARγ Ligand Assay

FAs binding to PPARγ were studied with a fluorescence polarization-based single-step PPARγ ligand screening assay (Cayman Chem. Co., Ann Arbor, MI, USA). This assay is based on the competition of free ligand in the samples or standards for the affinity binding site of PPARγ occupied by a probe conjugated to fluorescein. Finally, the polarization was quantified as milli-polarization units (mP). This assay was adapted to be performed in a microcuvette with a luminescence spectrometer (AMINCO-Bowman Series 2, Spectronic Unicam, Leeds, UK).

### Mitochondrial Membrane Potential Determination

Mitochondrial membrane depolarization (MMP) was measured through tetramethylrhodamine ethyl ester perclorate (TMRE) incorporation into active undifferentiated Caco-2 cell mitochondria. Carbonyl cyanide 3-chlorophenylhydrazone (CCCP) was used as a positive control for MMP. Cell cultures were treated with experimental compounds for 20 min and, after tripsinization, cell suspension was labeled with TMRE for 30 min. Finally, samples were analyzed in FC500 flow cytometer (Coulter Corporation) to measure mitochondrial depolarization or hyperpolarization.

### Data Analysis

The results are expressed as mean ± SEM. All data were compared by one-way ANOVA and the Student’s t-test, using SPSS software (SPSS Inc., Chicago, IL). Significance was taken as p < 0.05.

## Results

### Long-Chain Fatty Acids Affect Intestinal Epithelial Caco-2 Cell Growth

**Figure 1:**
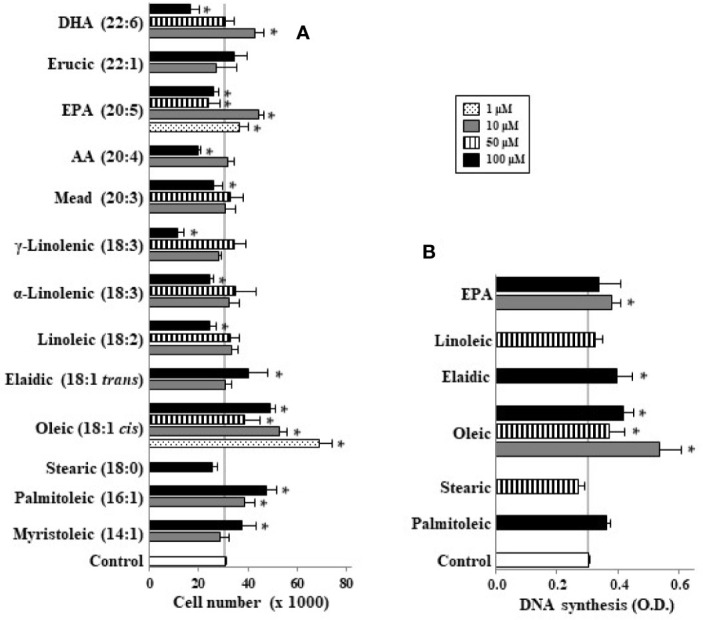
Effect of different LCFA on Caco-2 cell growth **(A)** and DNA synthesis **(B)** in absence of growth factors. Myristoleic, palmitoleic, stearic, oleic, elaidic, linoleic, α-linolenic, γ-linolenic, mead, AA, EPA, erucic, and DHA were incubated with Caco-2 cells at 1, 10, 50, and 100 µM (dot, gray, line, and black bars, respectively) without FBS for 48 h and were then counted or DNA synthesis measured by cell BrdU incorporation. Results are expressed like mean ± SEM (n = 8–30). *P < 0.05 *versus* control group (white bar, cells cultured without FBS).

**Figure 2:**
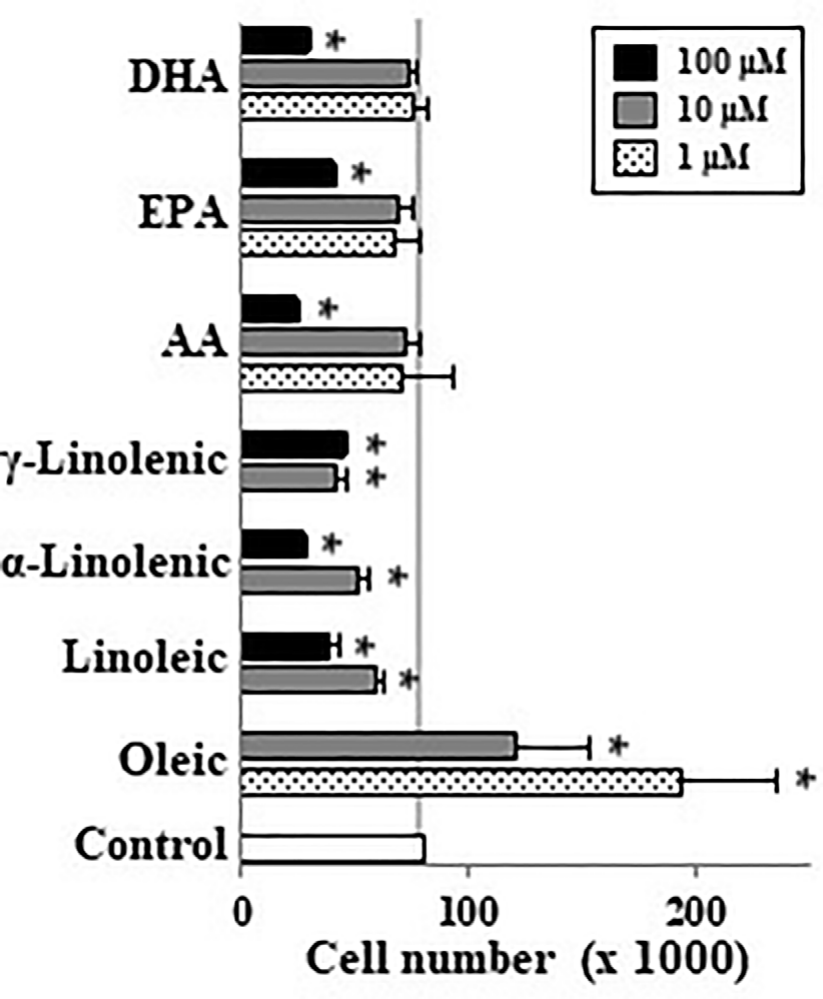
Effect of different LCFA on Caco-2 cell growth in presence of 10% FBS. Oleic, linoleic, α-linolenic, γ-linolenic, AA, EPA, and DHA were incubated with Caco-2 cells at 1, 10, and 100 µM (dot, gray, and black bars, respectively) in presence of 10% FBS for 48 h and were then counted. Results are expressed like mean ± SEM (n = 4–12). *P < 0.05 *vs.* control group (white bar, cells cultured with 10% FBS).

When undifferentiated Caco-2 cells were incubated with different LCFA in absence of FBS we observed that palmitoleic (10–100 µM), oleic (1–100 µM), myristoleic (100 µM), and elaidic (100 µM) acids as well as the lowest EPA and DHA concentration (10 µM) induced cell proliferation (**Figure 1A**). In contrast saturated FAs such as stearic did not have this effect. These mitogenic effects of the above-mentioned monounsaturated FAs (MUFAs) and FUFAs were confirmed in DNA synthesis analysis except for palmitoleic (**Figure 1B**). Interestingly, it can be also seen that highest concentrations of some FAs like linoleic, α and γ-linolenic, AA, EPA, and DHA promote a decrease in cell number below control cell group suggesting a cytotoxic or apoptotic action of these PUFAs (**Figure 1A**). Then, cells were also incubated with FBS to assess the proliferative or antiproliferative capacity of these FAs in the presence of growth factors. **Figure 2** shows how oleic acid (1–10 μM) enhanced the mitogenic effect induced by FBS whereas linoleic, α- and γ-linolenic, and AA decreased cell growth. At 1–10 μM EPA and DHA did not modify cell growth induced by FBS, whereas at 100 μM the antiproliferative action of these PUFA was confirmed. Thus, saturated LCFAs did not have any effect on Caco-2 cell growth, oleic acid was the most mitogenic MUFA assayed; and EPA as a representative PUFA, presented a dual effect on Caco-2 cell growth depending on concentration and the presence of FBS.

### Polyunsaturated Long-Chain Fatty Acids Induce Apoptosis in Caco-2 Cell Cultures

**Figure 3:**
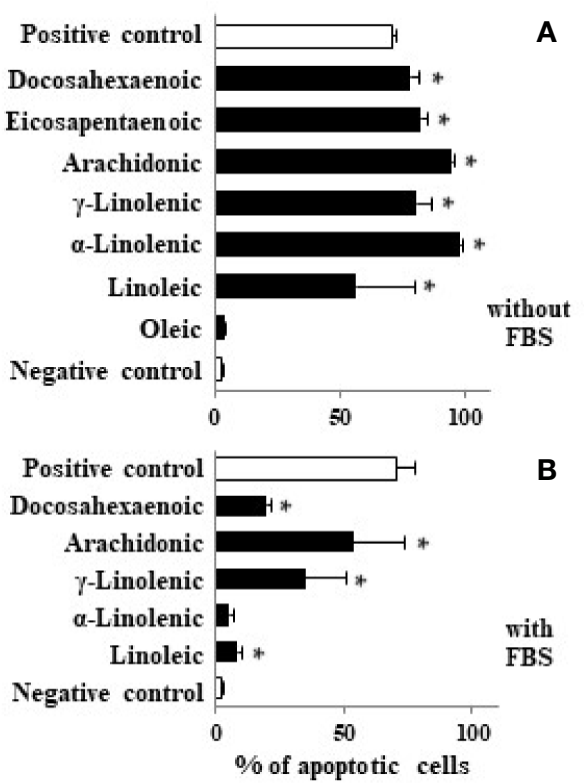
Effect of different LCFA on Caco-2 cell apoptosis. Caco-2 cells were incubated with stearic, oleic, linoleic, α-linolenic, γ-linolenic, AA, EPA, and DHA at 100 µM (black bars) in absence **(A)** or presence of FBS **(B)** for 48 h and DNA fragmentation was measured. Values are mean ± SEM (n = 3–8). *P < 0.05 *versus* negative control group (cells cultured in absence of FBS, white bar). As positive control we used staurosporine (1 µM) in presence of 10% FBS (white bar).

Considering that some LCFAs studied have antiproliferative effect at high concentrations, we analyzed their apoptotic activity by flow cytometry. Our results show that LCFAs such as α- and γ-linolenic, AA, EPA, and DHA induced apoptosis at high concentrations whereas oleic acid had no apoptotic activity (**Figure 3**). In addition, we measured the capacity of LCFAs to bind to PPARγ and to alter MMP (**Table 1**). We found that apoptotic LCFAs (α- and γ-linolenic, AA, EPA, and DHA) have affinity to PPARγ. Furthermore, AA, EPA, and DHA were capable to induce significant changes of MMP, both parameters implicated in the denominated intrinsic or mitochondrial pathway of apoptosis. Interestingly, low γ-linolenic, EPA, and DHA concentrations (10 μM) were not able to bind to PPARγ and to alter MMP (**Table 1**).

**Table 1:** Effect of LCFAs on PPARγ ligand assay and mitochondrial membrane potential variation.

Treatment	PPARγ bind (mP)	ΔMMP (au)
Vehicle	108 ± 9	21 ± 1.9
Rosiglitazone (10 μM)	43 ± 2*	ND
CCCP (5 μM)	ND	6 ± 0.5*
Staurosporine (1 μM)	ND	70 ± 4*
Oleic (100 μM)	109 ± 12	21 ± 1.6
Linoleic (100 μM)	98 ± 10	22 ± 0.7
α-Linolenic (100 μM)	76 ± 8*	24 ± 0.2
γ-Linolenic (10 μM)	91 ± 8	22 ± 0.7
γ-Linolenic (100 μM)	71 ± 16*	22 ± 0.9
AA (100 μM)	70 ± 8*	28 ± 1.9*
EPA (10 μM)	89 ± 7	23 ± 1,3
EPA (100 μM)	63 ± 15*	34 ± 1.3*
DHA (10 μM)	82 ± 7	25 ± 0.9
DHA (100 μM)	56 ± 3*	38 ± 0.7*

PPARγ binding was measured by fluorescence polarization and mitochondrial membrane potential variation (ΔMMP) was measured by flow cytometry as Material and Methods section described and was expressed as mP and arbitrary units (au), respectively. Results are expressed as mean ± SEM (n = 3–5). *P < 0.05 versus control group (vehicle). ND, non determined.

### The Mitogenic Effect of Oleic Acid and EPA Can Be Related With Cyclooxygenase and Lipoxygenase Metabolite Release

**Figure 4:**
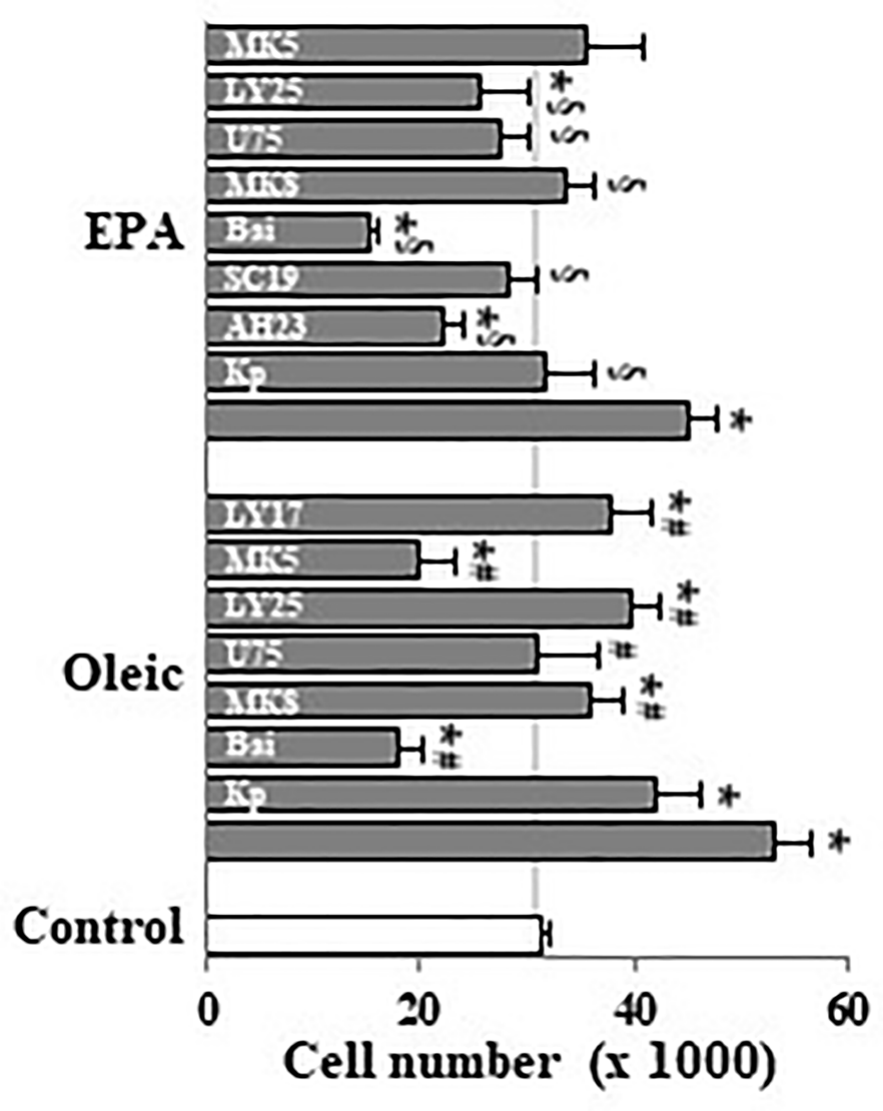
Effect of pharmacological modulation of AA cascade on Caco-2 cell growth induced by oleic and EPA. Caco-2 cells were incubated without FBS for 48 h with oleic or EPA at 10 µM in presence or absence of ketoprofen (Kp, 5 µM), AH23848 (AH23, 20 nM), SC19220 (SC19, 60 nM), baicalein (Bai, 25 µM), MK886 (MK8, 10 µM), U75302 (U75, 5 µM), LY25283 (LY25, 25 µM), MK571 (MK5, 25 µM), or LY171883 (LY17, 25 µM) and cells were counted. Results are expressed as mean ± SEM (n = 8–16). *P < 0.05 *vs.* control group (white bar, cells cultured without FBS), ^#^P < 0.05 *versus* oleic acid (10 µM), and ^§^P < 0.05 *versus* EPA (10 µM).

Undifferentiated Caco-2 cells expresses COX-1 and COX-2 (Martin-Venegas et al., 2006), 5-, 12-, and 15-LOX (Martin-Venegas et al., 2014) as well as BLT receptors (Martin-Venegas et al., 2014) and EP receptors (Rodriguez-Lagunas et al., 2010). Here, we observed that cell proliferation induced by oleic acid or EPA at 10 µM was reverted by MK886 (5-LOX inhibitor), baicalein (12-LOX inhibitor), U75302 (BLT_1_ antagonist), and LY255283 (BLT_1_ and BLT_2_ antagonist) (**Figure 4**). In addition, these experiments showed that ketoprofen (COX inhibitor), AH23838 (EP_4_ antagonist), SC19220 (EP_1_ antagonist) also reverted the mitogenic effect induced by EPA (**Figure 4**) whereas the mitogenic effect induced by oleic acid was similarly reverted by MK571 (cysteinyl leukotriene antagonist) and LY171883 (cysteinyl LT receptor antagonist) (**Figure 4**). All inhibitors and antagonists were used to optimal concentrations previously stablished (Cabral et al., 2013). The reversion of oleic/EPA mitogenic effects by AA cascade inhibitors and eicosanoid receptor antagonists suggests a role of oleic/EPA metabolites in the mitogenic effects induced by both FAs.

### Eicosanoids From EPA Such as PGE_3_ and 12-S-HEPE Are Mitogenic Whereas LTB_5_ Did Not Induce Caco-2 Cell Growth

**Figure 5:**
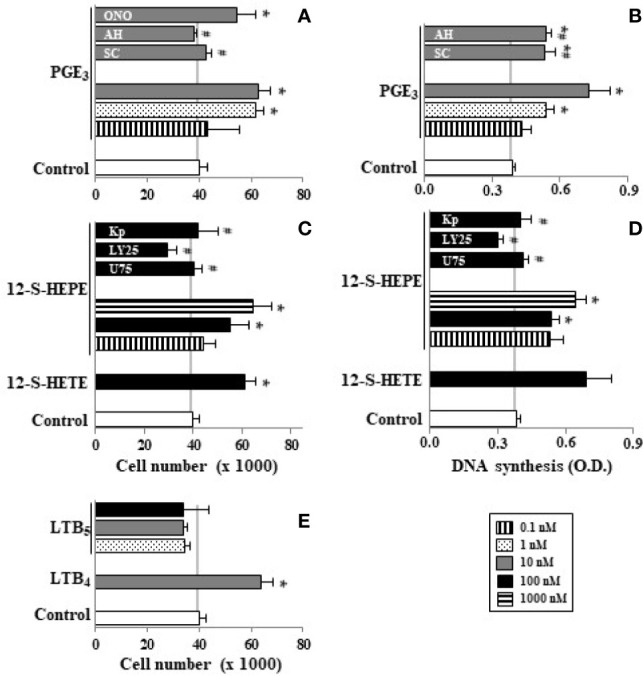
Effect of PGE_3_, 12-S-HEPE, and LTB_5_ on Caco-2 cell growth. Caco-2 cells were incubated for 48 h with PGE_3_ (0.1, 1, and 10 nM, line, dot, and gray bars, respectively) or PGE_3_ (10 nM) plus SC19220 (SC, 60 nM) or AH 23848 (AH, 20 nM) or ONO-AE3-240 (ON, 2 nM) or with 12-S-HEPE (10, 100, and 1000 nM, line, gray, and dot bars, respectively) or with 12-S-HEPE (100 nM, gray bar) plus U 75302 (U, 5 µM) or LY 255283 (LY, 25 µM) or ketoprofen (Kp, 5 µM). Cell cultures were also incubated with LTB_5_ (1, 10,and 100 nM, dot, gray, and black bars, respectively) or LTB_4_ (10 nM, gray bar) in absence of FBS. Finally, cells were then counted **(A, C, E)** and DNA synthesis was measured **(B, D)**. Data are expressed as means ± SEM of three to four experiments performed in triplicate. *P < 0.05 *vs.* Caco-2 cell cultures in the absence of FBS, ^#^P < 0.05 *vs.* cells incubated with 10 nM PGE_3_ or 12-S-HEPE.

EPA can be presented into cell membrane phospholipids (Lands et al., 1992), it is release by phospholipases (Nieves and Moreno, 2006), and it is metabolized by COX and LOX, giving rise to the 3-series of prostanoids, 5-series leukotrienes, and HEPEs (Dommels et al., 2002). Our results show that PGE_2_ concentrations in Caco-2 cell culture medium reach 1.52 ± 0.21 ng/ml (around 5 nM) whereas PGE_3_ was not detected. Interestingly, EPA (10 μM) supplementation of Caco-2 cell culture medium increases PGE_3_ synthesis (1.02 ± 0.13 ng/ml), whereas we observed the impairment of PGE2 (1.02 ± 0.09 ng/ml). In **Figures 5A, B** we observed that PGE_3_ progressively increased Caco-2 cell growth and DNA synthesis up to 10 nM in a similar way to PGE_2_. **Figures 5A, B** also show that Caco-2 cell growth and DNA synthesis induced by PGE_3_ was totally inhibited by an EP_1_ antagonist (SC19220) and by an EP_4_ antagonist (AH23838). However, an EP_3_ antagonist (ONO-AE3-240, 2nM) did not have any effect. Therefore, these results indicate that PGE_3_ acts through EP_1_ and EP_4_ receptors, but not through the EP_3_ receptor.

Our results also show that 12-S-HEPE (100–1000 nM) induced significant cell growth and DNA synthesis in Caco-2 cell cultures in the absence of growth factors, in a similar way to 12-S-HETE (100 nM). This mitogenic action of 12-S-HEPE was blocked by a COX inhibitor (ketoprofen), by a BLT_1_ antagonist (U75302), and by a BLT_1_ and BLT_2_ antagonist (LY255283) (**Figures 5C, D**).

Previously we observed that LTB_4_ has a mitogenic effect on Caco-2 cells (Cabral et al., 2013). However, our findings show that LTB_5_ derived from EPA did not induce proliferation in the range of 1–100 nM (**Figure 5E**).

Finally, we studied the capacity of PGE_3_ (10 nM) and 12-S-HEPE (100 nM) to phosphorylate pivotal elements in the cell signaling pathways implicated in the regulation of cell growth. Our findings show that PGE_3_ presents a similar pattern to PGE_2_. Thus, PGE_3_ could increase the phosphorylation of ERK 1/2, CREB, GSKβ, and p38α (**Table 2**), cell signaling pathways involved in cell growth. Similar effects were induced by 12-S-HEPE (**Table 2**).

**Table 2:** Effect of eicosanoids (PGE_3_ and 12-S-HEPE) and 10-HODE on cell signaling.

O.D. (450 nm)					
Pathway	Control	PGE_2_	PGE_3_	12-S-HEPE	10-HODE
AKT1	0.18 ± 0.04	0.25 ± 0.04	0.23 ± 0.03	0.19 ± 0.02	0.21 ± 0.03
AKT2	0.17 ± 0.02	0.19 ± 0.02	0.18 ± 0.03	0.21 ± 0.01	0.19 ± 0.02
P38α	0.42 ± 0.04	0.92 ± 0.04^*^	0.99 ± 0.05^*^	0.85 ± 0.03^*^	0.83 ± 0.04^*^
GSKβ	0.19 ± 0.03	0.56 ± 0.08^*^	0.52 ± 0.07^*^	0.48 ± 0.04^*^	0.50 ± 0.06^*^
CREB	0.21 ± 0.02	0.82 ± 0.11^*^	0.85 ± 0.12^*^	0.76 ± 0.13^*^	0.69 ± 0.10^*^
ERK 1/2	0.37 ± 0.03	1.06 ± 0.12^*^	0.99 ± 0.11^*^	0.95 ± 0.14^*^	0.89 ± 0.13^*^

Caco-2 cells were incubated with PGE_2_ or PGE_3_ (10 nM), 12-S-HEPE (100 nM), or 10-HODE (100 nM) for 5 or 15 min, cells were then collected and finally phosphorylated AKT1, AKT2, p38α, GSKβ, CREB, and ERK 1/2 were measured as described in the Material and Methods section and expressed as optical density (450 nm). Data are expressed as means ± SEM of two to four experiments performed in triplicate. ^*^P < 0.05 vs. Caco-2 cell cultures in the absence of FBS.

### 10-Hydroxy-8-Octadecenoic, LOX-Metabolite From Oleic Acid, Has Proliferative Effect in Caco-2 Cultures

**Figure 6:**
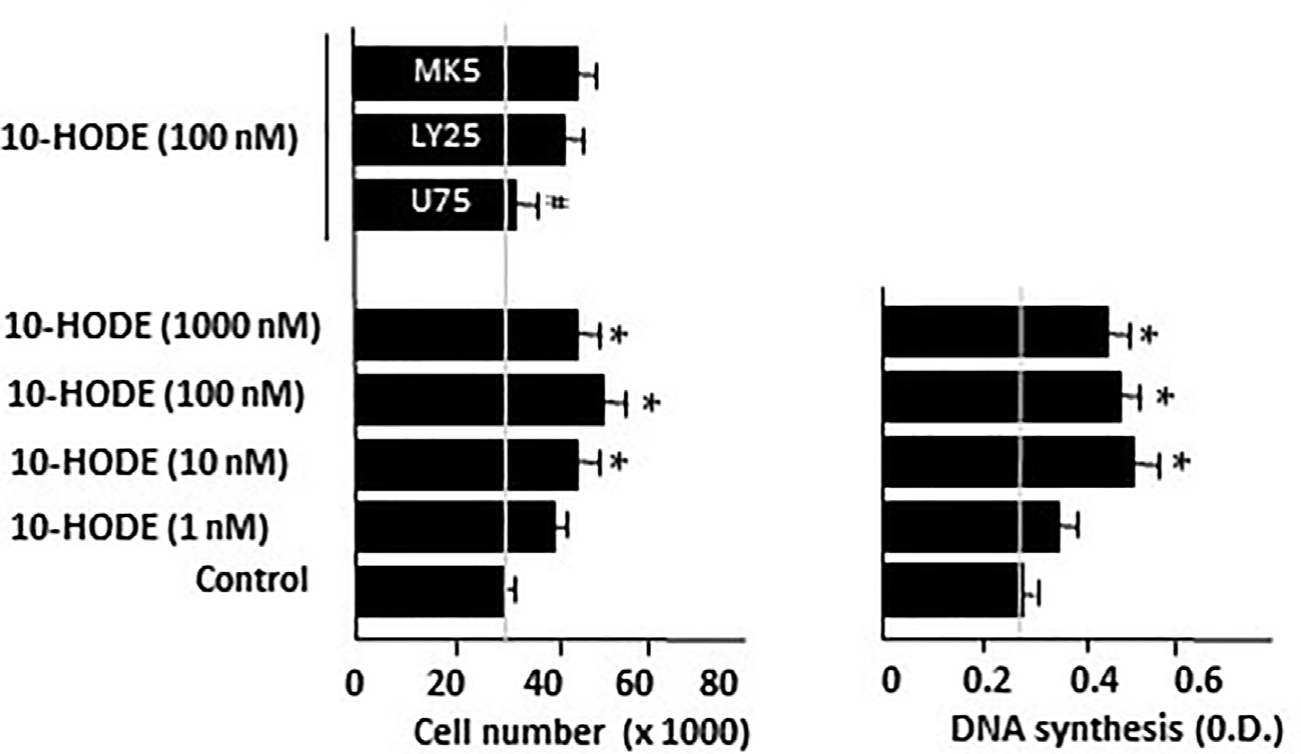
Effect of 10-HODE on Caco-2 cell growth and DNA synthesis. 10-HODE was incubated with Caco-2 cells without FBS in presence of U75302 (U75, 5 µM), LY25283 (LY25, 25 µM), or MK571 (MK5, 25 µM) for 48 h and then cells were counted or DNA synthesis assayed by cell BrdU incorporation. Results are expressed as mean ± SEM (n = 6–9) *P < 0.05 *vs* control group (cells cultured without FBS), ^#^P < 0.05 *vs* 10-HODE (100 nM), N.D., non determined.

Guerrero et al. (1997) and Clapp et al. (2006) reported the oxidation of oleic acid by bacterial and vegetal LOXs to produce 10-hydroxy-8-octadecenoic (10-HODE). Considering that undifferentiated Caco-2 cells have 5-, 12-, and 15-LOX activities (Cabral et al., 2013; Martin-Venegas et al., 2014), we hypothesized the implication of 10-HODE in the mitogenic effects of oleic acid. Our results show that 10-HODE acid induced significant cell growth and DNA synthesis in Caco-2 cell cultures in the absence of growth factors, in a similar way to oleic acid. This mitogenic action of 10-HODE was blocked by a BLT_1_ antagonist (U75302), but not by BLT_1_ and BLT_2_ antagonist (LY255283) nor by MK571, a cysteinyl leukotriene antagonist (**Figure 6**). Furthermore, we observed that 10-HODE was able to induce some mitogenic cell signaling pathways such as ERK 1/2, CREB, or p38α (**Table 2**).

## Discussion

Undigested carbohydrates are fermented in the large intestine to form short-chain fatty acids (SCFA) such as acetate, propionate, and butyrate. SCFAs and specially butyrate induces cellular differentiation and decrease proliferation of CRC-derived cell lines (Hass et al., 1997; Arantes et al., 2016). These effects may be related with the inhibition of histone deacetylase by DNA hypermethylation to promote cell differentiation (Boffa et al., 1978) and the inhibition of cell proliferation *via* p21, cyclin D1, and β-catenin pathway (Wang and Friedman, 1998). Thus, SCFAs have been reported to have important effects on intestinal epithelial cell growth/differentiation and consequently on CRC (Kles and Chang, 2006). However, there is a great debate about the role of saturated, monounsaturated, and polyunsaturated (ω-3 and ω-6) LCFAs in CRC, but few studies have been conducted on the potency of LCFAs and/or specific LCFA metabolites in modulating CRC cell line growth. Interestingly, our findings show that unsaturated LCFAs were mitogenic at physiological concentrations, whereas PUFAs such as EPA and DHA were mitogenic at low concentrations but at high concentrations might have the opposite effects. In contrast, saturated LCFAs did not show any effects of Caco-2 cell growth.

Our findings show that monounsaturated LCFAs such as palmitoleic and oleic acids are mitogenic in undifferentiated Caco-2 cell cultures being oleic acid the most active. This effect was also observed by the C_18:1_ *trans*, elaidic acid. These findings agree with Petrik et al. (2000), that reported that Apc^min/+^ mice feed with oleic acid present a high number of intestinal tumors, and recently, this mitogenic effect of oleic acid has also been observed in prostate cancer cell growth (Liotti et al., 2018). Interestingly, stearoyl-CoA desaturases expression and activity and the subsequent conversion of saturated LCFA to monounsaturated LCFA, were linked to CRC pathogenesis (Vargas et al., 2015; Igal, 2016), and consequently an accumulation of monounsaturated LCFAs. It is important to note that oleic 10–100 μM is reached in human plasma (Kopf and Schmitz, 2013).

Cancer cells are characterized by higher rates of lipid biosynthesis in addition to increased glycolysis and lactate production than those of normal cells (Currie et al., 2013) as well as high fatty acid uptake that require the expression of CD36, over-expressed in the majority of tumor tissues (Kuemmerle et al., 2011). This suggests that catabolism of FAs may be the dominant bioenergetics source in cancer cells and, thus, be an important fuel source for cell proliferation. Interestingly, our study reports data that suggest an alternative mechanism to explain the mitogenic action of oleic acid. Thus, our findings prompt that the mitogenic effect of oleic acid appears dependent on LOX pathway metabolism and the subsequent production of oleic acid metabolites. Clapp et al. (2006) reported that lipoxygenation of oleic acid gives allylic hydroperoxides followed by conversion to enones. In this sense, Cabral et al. (2013 and 2015) reported that LTs from AA and hydroxyoctadecanoid acids from linoleic acid induced Caco-2 cell growth. Thus, we can consider the possible implication of a LOX pathway oleic-derived metabolite in these events. Guerrero et al. (1997) reported that 10-HODE is biosynthesized from oleic acid by microbial oxidation that Clapp et al. (2006) attributed to LOX activity. The reversion of the mitogenic effects of oleic acid by LOX inhibitors suggest, by the first time in mammalian cells, that oleic acid is metabolized by LOXs and that 10-HODE or other oleic acid metabolite can be responsible, at least in part, of Caco-2 cell growth induced by this FA. Recently, we reported that LTs, HETEs, and hydroxyoctadecadienoic acids (Cabral et al., 2014; Cabral et al., 2015), with structural similarity to 10-HODE, have proliferative effects through BLT interaction and the subsequent COX-pathway activation. This study shows that 10-HODE from oleic acid was also able to induce proliferative cell signaling pathways and Caco-2 cell growth through common mechanisms with LTs and HETEs (Cabral et al., 2014; Cabral et al., 2015). Future studies should be designed to demonstrate 10-HODE synthesis by intestinal epithelial cells. Interestingly, Barone and co-workers (2014) observed a decrease in polyp number and polyp volume by olive oil diet. These apparent discrepancies put into consideration the fact that oleic acid and olive oil can exert different effect on CRC cell line growth considering that olive oil is a complex mix content bioactive compounds that can modulate oleic acid action. In this context, we recently reported that some minor compounds of extra virgin olive oil modulate the mitogenic effects of oleic acid on Caco-2 cells (Storniolo et al., 2019).

Dommels et al. (2002) reported that AA and EPA (10–100 µM) induced Caco-2 cell growth inhibition and cytotoxicity through peroxidation products generated during lipid peroxidation and COX activity. In agreement with these authors, we observed that other PUFAs such as α- and γ-linolenic and DHA were able to reduce cell number and that these findings were related with their capacity to induced apoptosis (mitochondrial membrane potential variation, DNA fragmentation) and binding to PPARγ, an event that Giros et al. (2009) did not consider involved in the apoptotic mechanism of ω-3 PUFAs on Caco-2 cells. These findings suggest a role of intrinsic pathway of apoptosis in the pro-apoptotic actions of these PUFAs. Interestingly, our findings indicate that although EPA as well as linoleic, α- and γ-linolenic, AA, and DHA are apoptotic at the highest concentration (around 100 µM) as was reported previously (Zhang et al., 2015; Storniolo, 2017), EPA and DHA have a mitogenic effect at 1–10 µM. In addition, we observed that this mitogenic effect of EPA is COX- and LOX-pathway dependent, suggesting that EPA metabolites could be involved in this event.

Experimental studies have shown that diets rich in fish oil significantly reduce the amount of AA present in membrane phospholipids (Mitjavila et al., 1996) and consequently the synthesis of AA metabolites such as PGE_2_ (Moreno et al., 2001), but increase the release of EPA metabolites. Even though the theory of formation of the 3-series PGs by EPA has been studied for decades, we still do not fully understand the role of EPA metabolites such as PGE_3_ in cancer cells (Yang et al., 2014). Here, we observed, for the first time, that PGE_3_ increased cell growth and DNA synthesis in non-differentiated intestinal epithelial cells at concentrations reached in Caco-2 cell cultures supplemented with EPA. Thus, PGE_3_ presents a proliferative action in a similar form to PGE_2_ at nM concentrations that could be reached in colonic tissue as consequence of the immune cell eicosanoid biosynthesis (Le Faouder et al., 2013). These findings are, apparently, in disagreement with Fan et al. (2014) who reported that PGE_3_ diminished the ability to support colonic stem cell expansion but using a non-physiological concentration (10 µM). Furthermore, we demonstrated that this PGE_3_ proliferative effect was a consequence of interaction with the PGE_2_ receptors EP_1_ and EP_4_, in agreement with their affinities (Moreno, 2017), and with a recent report showing similar effects of both PGs on the disruption of the intestinal epithelial barrier function (Rodriguez-Lagunas et al., 2013). Moreover, we observed that cell signaling pathways involved in the mitogenic action of PGE_3_ are like those involved in PGE_2_ action (Cabral et al., 2013), being p38α, CREB, and ERK 1/2 pathways involved in the mitogenic action of PGE_3_.

HETEs have mitogenic effect on different types of cells and are also involved in the pathogenesis of cancer (Moreno, 2009). Recently, 12-S-HETE was reported to have a proliferative effect on Caco-2 cells (Cabral et al., 2013). To our knowledge, this is the first study to show that 12-S-HEPE from EPA has similar effect on intestinal epithelial cell growth with the implication of ERK 1/2, CREB, or P38α pathways. No specific cellular receptors for HETEs/HEPEs have been identified to date. However, it has been reported that the binding of 12-HETE to the BLT_2_ receptor may be involved in its mitogenic action (Cabral et al., 2013). Here, we demonstrate that Caco-2 cell growth induced by 12-S-HEPE can be reverted by BLT_1_ and BLT_2_ antagonists and a COX inhibitor, which suggests that the 12-S-HEPE mitogenic action is, at least partly, due to PGs synthesis after 12-S-HEPE interaction with both BLT receptors; a mechanism previously described for LTB_4_ and 12-HETE (Cabral et al., 2013) and 13-R-hydroxyoctadecadienoic acid (44), compounds structurally related with 12-S-HEPE.

EPA administered to patients with Crohn’s disease (Ikehata et al., 1992) or ulcerative colitis (Salomon et al., 1990) increases the generation of LTB_5_ and the LTB_5_-LTB_4_ ratio, which were related with an improvement in these patients. In our study, LTB_5_ had no proliferative effect, while LTB_4_ significantly induced Caco-2 cell growth, findings in agreement with Bortuzzo et al. (1996) who found a lower affinity of LTB_5_ to the receptor of LTB_4_. Moreover, since the treatment with 5-LOX inhibitor or cysteinyl LT receptor antagonist reduced the mitogenic effect of EPA, these results indirectly suggest that 5-series cysteinyl LTs could also be involved, at least in part, in the mitogenic EPA effects, in our experimental conditions. There is little literature about the affinity of EPA derived LTs and cysteinyl LT receptors but Wallace and McKnight (1990) reported that LTC_5_ or LTD_5_ have biological activity although less potent than LTs derived from AA. Furthermore, although LTB_5_ did not have the mitogenic effect of its AA-derived partners, PGE_3_ and 12-S-HEPE have considerable mitogenic effects on intestinal epithelial Caco-2 cells and may be involved in cell proliferation induced by EPA. The role of COX and/or LOX pathway on cancer cell line growth has been reported using cell lines such as HT-29, HCA7, or LoVo (Hawcroft et al., 2010; Ganesh et al., 2012; Li et al., 2018). However, to our knowledge, it is the first time to report the effect of PGE_3_, LTB_5_, and 12-S-HEPE derived from EPA on epithelial cell growth. However, we believe that future research should analyze the role of EPA and EPA eicosanoids on non-transformed intestinal epithelial cell growth as well as intestinal epithelium development using *in vitro* and *in vivo* experimental models. Furthermore, it will be interesting to study the role of DHA metabolites to explain the proliferative effect of this PUFA at low concentrations.

**Figure 7:**
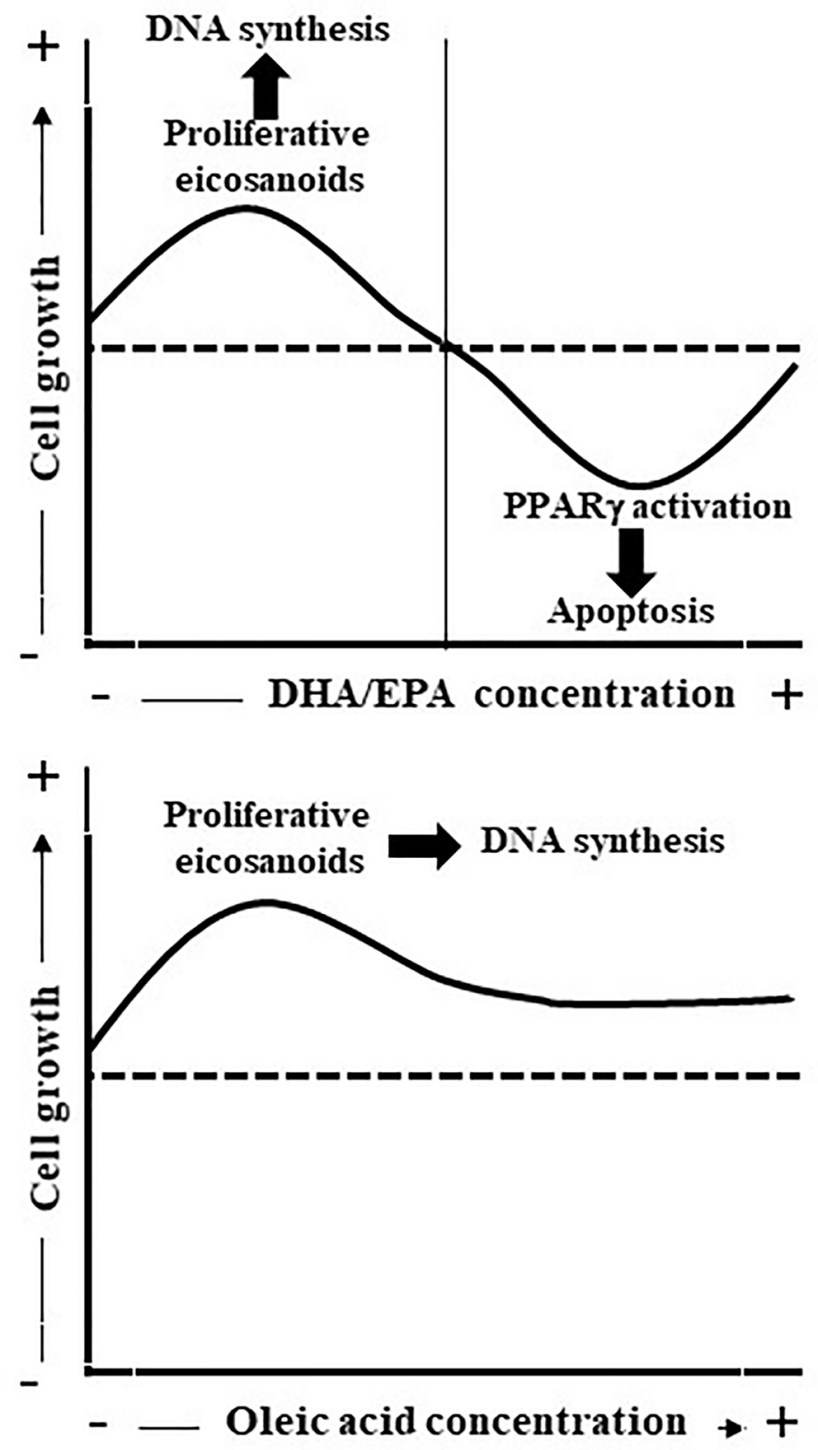
Scheme represents the effect of oleic acid and EPA/DHA on Caco-2 cell growth and apoptosis. Oleic acid is mitogenic probably through LOX pathway metabolite synthesis whereas EPA/DHA present a dual effect on Caco-2 cell proliferation. Low EPA concentrations (up to 10 µM) induce Caco-2 cell growth as consequence, at least in part, of PGE_3_ and 12-HEPE release whereas high EPA concentrations inhibit Caco-2 cell growth and induce apoptosis through binding to PPARγ.

In conclusion, the results obtained herein demonstrated that oleic acid is a potent mitogenic factor to undifferentiated Caco-2 cells probably through LOX pathway metabolite such as 10-HODE whereas PUFAs such as EPA or DHA have a dual behavior effect on Caco-2 cell growth depending on the FA concentration (**Figure 7**). A high concentration of EPA/DHA induced apoptosis, a process related with its binding to PPARγ. Meanwhile, low EPA concentration induced Caco-2 cell proliferation that could be related to the synthesis of mitogenic eicosanoids such as PGE_3_ and 12-HEPE and the subsequent induction of mitogenic cell signaling pathways. Obviously, these conclusions do not exclude a direct effect of LCFA (oleic acid and EPA) or LCFA metabolites on Caco-2 cell growth through bind to GPR40 and the subsequent cell signaling activation involved in cell proliferation (Hass et al., 1997; Liu et al., 2013) or GPR120 that is abundantly expressed in intestine (Hirasawa et al., 2005; Wu et al., 2013), it is activated by LCFAs (Hirasawa et al., 2005) and is involved in the mitogenic effects of PUFAs (Hass et al., 1997). Thus, this study contributes to open new perspectives to understand the complicated relationship between fat ingest and CRC.

## Data Availability Statement

The raw data supporting the conclusions of this article will be made available by the authors, without undue reservation, to any qualified researcher.

## Author Contributions

RM-V and JJM designed experiments and CES, MC, and MAB performed these experiments. CES, RM-V, and JJM carried out the data analysis and RM-V and JJM wrote the manuscript. All authors contributed to the article and approved the submitted version.

## Funding

This work was supported by the Ministerio de Ciencia e Innovación (BFU2007-61727/BFI, AGL2013-49083-C3-1-R and AGL2016-75329R), by Generalitat de Catalunya (2009SGR438) and by CIBEROBN (Instituto de Salud Carlos III).

## Conflict of Interest

The authors declare that the research was conducted in the absence of any commercial or financial relationships that could be constituted as a potential conflict of interest.

## Abbreviations

AA, arachidonic acid; CCCP, carbonyl cyanide 3-chlorophenylhydrazine; COX, cyclooxygenase; CRC, colorectal cancer; HEPE, hydroxyeicosapentaenoic acid; LCFA, long-chain fatty acids; LOX, lipoxygenase; LT, leukotriene; PG, prostaglandin; SCFA, short-chain fatty acids; TMRE, tetramethylrhodamine ethyl ester.

## References

[B1] Arantes E. L. Dragano N. Ramalho A. Vitorino D. de Souza G. F. Lima M. H. (2016). Topical docosahexaenoic acid (DHA) accelerates skin wound healing in rats and activates GPR120. Biol. Res. Nursery 18, 411–419. 10.1177/10998004155621617 26747719

[B2] Barone M. Notarnicola M. Caruso M. G. Scavo M. P. Viggiani M. T. Tutino V. (2014). Olive oil and omega-3 polyunsaturated fatty acids suppress intestinal polyp growth by modulating the apoptotic process in ApcMin/+ mice. Carcinogenesis 35, 1613–1619. 10.1093/carcin/bgu068 24632492

[B3] Boffa L. C. Vidali G. Mann R. S. Allfrey V. G. (1978). Suppression of histone deacetylation in vivo and in vitro by sodium butyrate. J. Biol. Chem. 253, 3364–3366. 649576

[B4] Bortuzzo C. Hanif R. Kashfi K. Staiano-Coico L. Shiff S. J. Rigas B. (1996). The effect of leukotrienes B and selected HETEs on the proliferation of colon cancer cells. Biochim. Biophys. Acta Lipids Lipid Metab. 1300, 240–246. 10.1016/0005-2760(96)00003-3 8679690

[B5] Cabral M. Martín-Venegas R. Moreno J. J. (2013). Role of arachidonic acid metabolites on the control of non-differentiated intestinal epithelial cell growth. Int. J. Biochem. Cell. Biol. 45, 1620–1628. 10.1016/j.biocel.2013.05.009 23685077

[B6] Cabral M. Martin-Venegas R. Moreno J. J. (2014). Differential cell growth/apoptosis behavior of 13-hydroxyoctadecadienoic acid enantiomers in a colorectal cancer cell line. Am. J. Physiol. Gastrointest. Liver Physiol. 307, G664–G671. 10.1152/ajpgi.00064.2014 25035111

[B7] Cabral M. Martin-Venegas R. Moreno J. J. (2015). Leukotriene D4-inducedCaco-2 cell proliferation is mediated by prostaglandin E2 synthesis. Physiol. Rep. 3, e12417. 10.14814/phy2.12417 26216432 PMC4552517

[B8] Chao A. Thun M. J. Connell C. J. McCullough M. L. Jacobs E. J. Flanders W. D. (2005). Meat consumption and risk of colorectal cancer. JAMA 293, 172–182. 10.1001/jama.293.2.172 15644544

[B9] Clapp C. H. Strulson M. Rodriguez P. C. Lo R. Novak M. J. (2006). Oxygenation of monounsaturated fatty acids by soybean lipoxygenase-1: Evidence for transient hydroperoxide formation. Biochemistry 45, 15884–15892. 10.1021/bi0619425 17176111

[B10] Clinton S. K. Giovannucci E. L. Hursting S. D. (2020). The World Cancer Research Fund/American Institute for Cancer Research Third Expert Report on Diet, Nutrition, Physical Activity, and Cancer: Impact and Future Directions. J. Nutr. 150, 663–667. 10.1093/jn/nxz268 31758189 PMC7317613

[B11] Cockbain A. J. Toogood G. J. Hull M. A. (2012). Omega-3 polyunsaturated fatty acids for the treatment and prevention of colorectal cancer. Gut 61, 135–149. 10.1136/gut.2010.233718 21490374

[B12] Currie E. Schulze A. Zechner R. Walther T. C. Farese R. V. (2013). Cellular fatty acid metabolism and cancer. Cell Metab. 18, 153–161. 10.1016/j.cmet.2013.05.017 23791484 PMC3742569

[B13] Daniel C. R. McCullough M. L. Patel R. C. Jacobs E. J. Flanders W. D. Thun M. J. (2009). Dietary intake of omega-6 and omega-3 fatty acids and risk of colorectal cancer in a prospective cohort of U.S. men and women. Cancer Epidemiol. Biomarkers Prev. 18, 516–525. 10.1158/1055-9965.EPI-08-0750 19190143

[B14] Dommels Y. E. Alink G. M. Van Bladeren P. J. Van Ommen B. (2002). Dietary n-6 and n-3 polyunsaturated fatty acids and colorectal carcinogenesis: Results from cultured colon cells, animal models and human studies. Environ. Toxicol. Pharmacol. 11, 297–308. 10.1016/S1382-6689(02)00006-6 21782613

[B15] Escrich E. Moral R. Grau L. Costa I. Solanas M. (2007). Molecular mechanisms of the effects of olive oil and other dietary lipids on cancer. Mol. Nutr. Food Res. 51, 1279–1292. 10.1002/mnfr.200700213 17879998

[B16] Fan Y. Y. Davidson L. A. Callaway E. S. Goldsby J. S. Chapkin R. S. (2014). Differential effects of 2-and 3-series E-prostaglandins on in vitro expansion of Lgr5+ colonic stem cells. Carcinogenesis 35, 606–612. 10.1093/carcin/bgt412 24336194 PMC3941743

[B17] Ganesh R. Marks D. J. Sales K. Winslet M. C. Seifalian A. M. (2012). Cyclooxygenase/lipoxygenase shunting lowers the anti-cancer effect of cyclooxygenase-2 inhibition in colorectal cancer cells. World J. Surg. Oncol. 10, 200. 10.1186/1477-7819-10-200 23013454 PMC3527267

[B18] Giovannucci E. Goldin B. (1997). The role of fat, fatty acids, and total energy intake in the etiology of human colon cancer. Am. J. Clin. Nutr. 66, 1564S–1571S. 10.1093/ajcn/66.6.1564S 9394716

[B19] Giros A. Grzybowski M. Sohn V. R. Pons E. Fernandez-Morales J. Xicola R. M. (2009). Regulation of colorectal cancer cell apoptosis by the n-3 polyunsaturated fatty acids docosahexaenoic and eicosapentaenoic. Cancer Prev. Res. 1, 732–742. 10.1158/1940-6207.CAPR-08-0197 PMC3793343 19638488

[B20] Guerrero A. Casals I. Busquests M. Leon Y. Manresa A. (1997). Oxydation of oleic acid to €-10-hydroperoxy-8-octadecenoic and €-10-hydroxy-8-octadecenoic acids by Pseudomonas sp. 42A2 Biochim. Biophys. Acta 1347, 75–81. 10.1016/S0005-2760(97)00056-8 9233689

[B21] Hass R. Busche R. Luciano L. Reale E. Engelhardt W. V. (1997). Lack of butyrate is associated with induction of Bax and subsequent apoptosis in the proximal colon of guinea pig. Gastroenterology 112, 875–881. 10.1053/gast.1997.v112.pm9041249 9041249

[B22] Hawcroft G. Loadman P. M. Belluzzi A. Hull M. A. (2010). Effect of eicosapentaenoic acid on E-type prostaglandin synthesis and EP4 receptor signaling in human colorectal cancer cells. Neoplasia 12, 618–627. 10.1593/neo.10388 20689756 PMC2915406

[B23] Hirasawa A. Tsumaya K. Awaji T. Katsumara S. Adachi T. Yamada M. (2005). Free fatty acids regulate gut incretin glucagon-like peptide-1 secretion through GPR120. Nat. Med. 11, 90–94. 10.1038/nm1168 15619630

[B24] Igal R. A. (2016). Stearoyl CoA desaturase-1: New insights into a central regulator of cancer metabolism. Biochim. Biophys. Acta 1861, 1865–1880. 10.1016/j.bbalip.2016.09.009 27639967

[B25] Ikehata A. Hiwatashi N. Kinouchi Y. Yamazaki H. Kumagai Y. Ito K. (1992). Effect of intravenously infused eicosapentaenoic acid on the leukotriene generation in patients with active Crohn’s disease. Am. J. Clin. Nutr. 56, 938–942. 10.1093/ajcn/56.5.938 1329484

[B26] Kles K. A. Chang E. B. (2006). Short-chain fatty acids impact on intestinal adaptation, inflammation, carcinoma, and failure. Gastroenterology 130, S100–S105. 10.1053/j.gastro.2005.11.048 16473056

[B27] Kopf T. Schmitz G. (2013). Analysis of non-esterified fatty acids in human samples by solid-phase-extraction and gas chromatography/mass spectrometry. J. Chromatogr. B. Analyt. Technol. Biomed. Life Sci. 938, 22–26. 10.1016/j.jchromb.2013.08.016 24036177

[B28] Kuemmerle N. B. Rysman E. Lombardo P. S. Flanagan A. J. Lipe B. C. Wells W. A. (2011). Lipoprotein lipase links dietary fat to solid tumor cell proliferation. Mol. Cancer Ther. 10, 427–436. 10.1158/1535-7163.MCT-10-0802 21282354 PMC3074101

[B29] Lands W. B. Libelt B. Morris A. Kramer N. C. Prewitt T. E. Schmeisser D. (1992). Maintenance of lower proportions of (n-6) eicosanoid precursors in phospholipids of human plasma in response to added dietary (n-3) fatty acids. Biochim. Biophys. Acta 1180, 147–162. 10.1016/0925-4439(92)90063-S 1463766

[B30] Larsson S. C. Kumlin M. Ingelman-Sundberg M. Wolk A. (2004). Dietary long-chain n-3 fatty acids for the prevention of cancer: a review of potential mechanisms. Am. J. Clin. Nutr. 79, 935–945. 10.1093/ajcn/79.6.935 15159222

[B31] Le Faouder P. Baillif V. Spreadbury I. Motta J. P. Rousset P. Chene G. (2013). LC-MS/MS method for rapid and concomitant quantification of pro-inflammatory and pro-resolving polyunsaturated fatty acid metabolites. J. Chromatogr. B. 932, 123–133. 10.1016/j.chromb.2013.06.014 23831705

[B32] Li Y. Shi J. Qi S. Zhang J. Peng D. Chen Z. (2018). IL-33 facilitates proliferation of colorectal cancer dependent on COX2/PGE2. J. Exp. Clin. Cancer Res. 17, 196. 10.1186/s13046-018-0839-7 PMC6098640 30119635

[B33] Liotti A. Cosimato V. Mirra P. Cali G. Conza D. Secondo A. (2018). Oleic acid promotes prostate cancer malignant phenotype via the G protein-coupled receptor FFA1/GPR40. J. Cell. Physiol. 233, 7367–7378. 10.1002/jcp26572 29663374 PMC13482116

[B34] Liu Z. Xiao Y. Yuan Y. Zhang X. Qin C. Xie J. (2013). Effects of oleic acid on cell proliferation through an integrin-linked kinase signaling pathway in 786-O renal cell carcinoma cells. Oncol. Lett. 5, 1395–1399. 10.3892/ol.2013.1160 23599801 PMC3629253

[B35] Martín-Venegas R. Roig-Pérez S. Ferrer R. Moreno J. J. (2006). Arachidonic acid cascade and epithelial barrier function during Caco-2 cell differentiation. J. Lipid Res. 47, 1416–1423. 10.1194/jlr.M500564-JLR200 16585783

[B36] Martin-Venegas R. Jáuregui O. Moreno J. J. (2014). Liquid chromatography-tandem mass spectrometry analysis of eicosanoids and related compounds in cell models. J. Chromatogr. B. 964, 41–49. 10.1016/j.jchromb.2014.05.024 24932539

[B37] Meyerhardt J. A. Niedzwiecki D. Hollis D. Saltz L. B. Hu F. B. Mayer R. J. (2007). Association of dietary patterns with cancer recurrence and survival in patients with stage III colon cancer. JAMA 298, 754–764. 10.1001/jama.298.7.754 17699009

[B38] Mitjavila M. T. Rodríguez M. C. Sáiz M. P. Lloret S. Moreno J. J. (1996). Effect of degree of unsaturation in dietary fatty acids on arachidonic acid mobilization by peritoneal macrophages. Lipids 31, 661–666. 10.1007/BF02523839 8784749

[B39] Moreno J. J. Carbonell T. Sánchez T. Miret S. Mitjavila M. T. (2001). Olive oil decreases both oxidative stress and the production of arachidonic acid metabolites by the prostaglandin G/H synthase pathway in rat macrophages. J. Nutr. 131, 2145–2149. 10.1093/jn/131.8.2145 11481409

[B40] Moreno J. J. (2005). Arachidonic acid cascade enzyme inhibition and cancer. Curr. Enzyme Inhibition 2, 131–145. 10.2174/1573408054022261

[B41] Moreno J. J. (2009). New aspects of the role of hydroxyeicosatetraenoic acids in cell growth and cancer development. Biochem. Pharmacol. 77, 1–10. 10.1016/j.bcp.2008.07.033 18761324

[B42] Moreno J. J. (2017). Eicosanoid receptors: Targets for the treatment of disrupted intestinal epithelial homeostasis. Eur. J. Pharmacol. 796, 7–19. 10.1016/j.ejphar.2016.12.004 27940058

[B43] National Cancer Institute National cancer institute surveillance epidemiology and end results. Finding Statistics. Available at: https://seer.cancer.gov/statfacts/html/colorect.html (Accessed May 22, 2017).

[B44] Nieves D. Moreno J. J. (2006). Effect of arachidonic and eicosapentaenoic acid metabolism on RAW 264.7 macrophage proliferation. J. Cell. Physiol. 208, 428–434. 10.1002/jcp.20678 16646088

[B45] Norat T. Bingham S. Ferrari P. Slimani N. Jenab M. Mazuir M. (2005). Meat, fish, and colorectal cancer risk: The European prospective investigation into cancer and nutrition. J. Nat. Cancer Inst. 97, 906–916. 10.1093/jnci/dji164 15956652 PMC1913932

[B46] Petrik M. B. McEntee M. F. Johnson B. T. Obukowicz M. G. Whelan J. (2000). Highly unsaturated (n-3) fatty acids, but not α-linolenic, conjugated linoleic or γ-linolenic acids, reduce tumorigenesis in Apc(Min/+) mice. J. Nutr. 130, 2434–2443. 10.1093/jn/130.10.2434 11015469

[B47] Rodriguez-Lagunas M. J. Martín-Venegas R. Moreno J. J. Ferrer R. (2010). PGE_2_ promotes Ca^2+^-mediated epithelial barrier disruption through EP1 and EP4 receptors in Caco-2 cell monolayers. Am. J. Physiol. Cell Physiol. 299, 324–334. 10.1152/ajpcell.00397.2009 20484658

[B48] Rodríguez-Lagunas M. J. Storniolo C. E. Ferrer R. Moreno J. J. (2013). 5-Hydroxyeicosatetraenoic acid and leukotriene D4 increase intestinal epithelial paracellular permeability. Int. J. Biochem. Cell. Biol. 45, 1318–1326. 10.1016/j.biocel.2013.04.005 23583294

[B49] Rosignoli P. Fabiani R. De Bartolomeo A. Fuccelli R. Pelli M. A. Morozzi G. (2008). Genotoxic effect of bile acids on human normal and tumour colon cells and protection by dietary antioxidants and butyrate. Eur. J. Nutr. 47, 301–309. 10.1007/s00394-008-0725-8 18685914

[B50] Salomon P. Kornbluth A. A. Janowitz H. D. (1990). Treatment of ulcerative colitis with fish oil n–3-omega-fatty acid: an open trial. J. Clin. Gastroenterol. 12, 157–161. 10.1097/00004836-199004000-00009 2109004

[B51] Sasazuki S. Inoue M. Iwasaki M. Sawada N. Shimazu T. Yamaji T. (2011). Intake of n-3 and n-6 polyunsaturated fatty acids and development of colorectal cancer by subsite: Japan Public Health Center-based prospective study. Int. J. Cancer 129, 1718–1729. 10.1002/ijc.25802 21120874

[B52] Shen X. J. Zhou J. D. Dong J. Y. Ding W. Q. Wu J. C. (2012). Dietary intake of n-3 fatty acids and colorectal cancer risk: a meta-analysis of data from 489 000 individuals. Br. J. Nutr. 108, 1550–1556. 10.1017/S0007114512003546 22906228

[B53] Smith W. L. (2005). Cyclooxygenases, peroxide tone and the allure of fish oil. Curr. Opin. Cell Biol. 17, 174–182. 10.1016/j.ceb.2005.02.005 15780594

[B54] Song M. Chan A. T. Fuchs C. S. Ogino S. Hu F. B. Mozaffarian D. (2014). Dietary intake of fish, ω-3 and ω-6 fatty acids and risk of colorectal cancer: A prospective study in U.S. men and women. Int. J. Cancer 135, 2413–2423. 10.1002/ijc.28878 24706410 PMC4159425

[B55] Storniolo C. E. Martínez-Hovelman N. Martínez-Huélamo M. Lamuela-Raventos R. M. Moreno J. J. (2019). Extra Virgin Olive Oil Minor Compounds Modulate Mitogenic Action of Oleic Acid on Colon Cancer Cell Line. J. Agric. Food Chem. 67, 11420–11427. 10.1021/acs.jafc.9b04816 31545039

[B56] Storniolo C. E. (2017). Efecto de componentes de la Dieta Mediterránea sobre la cascada del ácido araquidónico y la proliferación de células epiteliales intestinales (University of Barcelona: Doctoral Thesis).

[B57] Ullman T. A. Itzkowitz S. H. (2011). Intestinal inflammation and cancer. Gastroenterology 140, 1807–1816. 10.1053/j.gastro.2011.01.057 21530747

[B58] Vargas T. Moreno-Rubio J. Herranz J. Cejas P. Molina S. González-Vallinas M. (2015). ColoLipidGene: signature of lipid metabolism-related genes to predict prognosis in stage-II colon cancer patients. Oncotarget 6, 7348–7363. 10.18632/oncotarget.3130 25749516 PMC4466690

[B59] Wallace J. L. McKnight G. W. (1990). Comparison of the damage-promoting effects of leukotrienes derived from eicosapentaenoic acid and arachidonic acid on the rat stomach. J. Exp. Med. 171, 1827–1832. 10.1084/jem.171.5.1827 2332738 PMC2187910

[B60] Wang J. Friedman E. A. (1998). Short-chain fatty acids induce cell cycle inhibitors in colonocytes. Gastroenterology 114, 940–946. 10.1016/S0016-5085(98)70313-0 9558282

[B61] Williams C. D. Satia J. A. Adair L. S. Stevens J. Galanko J. Keku T. O. (2010). Associations of red meat, fat, and protein intake with distal colorectal cancer risk. Nutr. Cancer 62, 701–709. 10.1080/01635581003605938 20661817 PMC3023148

[B62] World Cancer Research Fund / American Institute for Cancer Research (2011).

[B63] World Health Organization http://www.who.int/dietphysicalactivity/publications/trs916/kit/en/.

[B64] Wu Q. Wang H. Zhao X. Shi Y. Jin M. Wan B. (2013). Identification of G-protein-coupled receptor 120 as a tumor-promoting receptor that induces angiogenesis and migration in human colorectal carcinoma. Oncogene 32, 5541–5550. 10.1038/onc.2013.264 23851494

[B65] Yang P. Jiang Y. Fischer S. M. (2014). Prostaglandin E3 metabolism and cancer. Cancer Lett. 348, 1–11. 10.1016/j.canlet.2014.03.010 24657656 PMC4366418

[B66] Yusof A. S. Isa Z. M. Shah S. A. (2012). Dietary patterns and risk of colorectal cancer: a systematic review of cohort studies, (2000-2011). Asian Pacific J. Cancer Prev. 13, 4713–4717. 10.7314/APJCP.2012.13.9.4713 23167408

[B67] Zhang C. Yu H. Shen Y. Ni X. Shen S. Das U. N. (2015). Polyunsaturated fatty acids trigger apoptosis of colon cancer cells through a mitochondrial pathway. Arch. Med. Sci. 11, 1081–1094. 10.5114/aoms.2015.54865 26528354 PMC4624753

